# Preventive, personalized medicine at the level of key regulatory proteins: updates on activity-dependent neuroprotective protein (ADNP) as a case study

**DOI:** 10.1186/1878-5085-5-S1-A97

**Published:** 2014-02-11

**Authors:** Illana Gozes

**Affiliations:** 1 The Adams Super Center for Brain Studies; The Lily and Avraham Gildor Chair for the Investigation of Growth Factors; The Elton Laboratory for Neuroendocrinology; Department of Human Molecular Genetics and Biochemistry, Sagol School of Neuroscience, Sackler Faculty of Medicine, Tel Aviv University, Tel Aviv 69978, Israel

## 

Natural ageing, neurodegenerative diseases, acute head (brain) injury and stroke as well as mental disorders are associated with nerve cell damage and death. We have discovered activity-dependent neurotrophic factor (ADNP) that is essential for brain formation, regulating >400 important genes, including the major risk gene for Alzheimer's disease, apolipoprotein E. ADNP constitutes a part of complex essential for proper gene activity [1]. In men, the ADNP gene is mutated in autism, deregulated in terms of brain expression in schizophrenia [2] and significantly reduced in the blood of patients with neurodegenerative disease such as Alzheimer's disease [3] and multiple sclerosis. New results indicate the potential of ADNP monitoring as indicator for disease progression, paving the path to preventive treatment with ADNP replacement therapies. In this respect, the ADNP peptide derivative NAP (davunetide) has shown neuroprotection in multiple animal models of neurodegeneration (protecting against axonal transport deficits) [4] as well as in a recent schizophrenia clinical trial [5]. It is thus of major interest to further investigate ADNP as a predictive marker and ADNP replacement as a personalized, preventive, protecting therapy against neurodegeneration.

## Support

AMN Foundation, Adams Super Center for Brain Studies, Canadian Friends of Tel Aviv University, Montreal Circle of Friends and the Adams Family.
